# Support Needs for Canadian Health Providers Responding to Disaster: New Insights from a Grounded Theory Approach

**DOI:** 10.1371/currents.dis.79dc64efd8e2af3488a126afa464c5d7

**Published:** 2015-07-01

**Authors:** Christine Fahim, Tracey L. O'Sullivan, Dan Lane

**Affiliations:** Interdisciplinary School of Health Sciences, University of Ottawa, Ottawa, Ontario, Canada; Interdisciplinary School of Health Sciences and Telfer School of Management, University of Ottawa, Ottawa, Ontario, Canada; Telfer School of Management, University of Ottawa, Ottawa, Ontario, Canada

## Abstract

Introduction: An earlier descriptive study exploring the various supports available to Canadian health and social service providers who deployed to the 2010 earthquake disaster in Haiti, indicated that when systems are compromised, professionals are at physical, emotional and mental risk during overseas deployment. While these risks are generally well-identified, there is little literature that explores the effectiveness of the supports in place to mitigate this risk. This study provides evidence to inform policy development regarding future disaster relief, and the effectiveness of supports available to responders assisting with international disaster response.

Methods: This study follows Strauss and Corbin’s 1990 structured approach to grounded theory to develop a framework for effective disaster support systems. N=21 interviews with Canadian health and social service providers, who deployed to Haiti in response to the 2010 earthquake, were conducted and analyzed. Resulting data were transcribed, coded and analysed for emergent themes.

Results and Discussion: Three themes were identified in the data and were used to develop the evolving theory. The interview data indicate that the experiences of responders are determined based on an interaction between the individual’s ‘lens’ or personal expectations, as well as the supports that an organization is able to provide. Therefore, organizations should consider the following factors: experience, expectations, and supports, to tailor a successful support initiative that caters to the needs of the volunteer workforce.

## Introduction

The 2010 earthquake in Haiti was one of the most devastating large-scale natural disasters of recent times. The earthquake destroyed hospitals, clinics, and other infrastructure, leaving many Haitian health and social service workers buried in the rubble.^1^^,^ 2 With the extent of the disaster, and majority of Haiti’s remaining social services directed at providing essential services, such as food, shelter and housing to their citizens, there were few resources left for medical and psychological care. Therefore, in order to cope with the medical demands of its people, Haiti relied heavily on international relief.^3^^,^ 4 The literature indicates that healthcare workers volunteering to assist with large-scale emergencies and natural disasters generally feel unprepared to respond effectively. 5 Response organizations must therefore ensure that their volunteers are not put at physical, emotional, or mental health risk.^6^ This requires an exploration of the supports currently in place for health and social service workers and an identification of any perceived gaps in the system. Designing an effective system for emergency preparedness requires a bottom-up approach, with input from professionals involved in the front-line response.

The purpose of this research is to understand and evaluate the lived experiences of health and social service workers deployed to Haiti in response to the 2010 earthquake. Using this data, we will develop a theoretical model highlighting the support systems required to ensure physical, emotional and psychological protection for volunteers responding to international disasters.

## Methodology

Twenty-one Canadian health and social service providers who responded to the disaster in Haiti were interviewed by the primary author. Participants for this study were recruited using maximum variation, purposeful sampling. To guarantee a range of experiences were represented in the interviews, participants were intentionally selected to reflect various career backgrounds, years of experience, length of deployment, and date of departure. A list of participant characteristics is included in Table 1. Each key informant was asked to participate in a single audio-recorded interview, approximately one hour in duration. This study was approved by an academic Research Ethics Board.

Participants were made aware that their information would remain confidential, and were required to sign a form of informed consent prior to the start of the interview. The participants were asked questions, according to a semi-structured interview guide, regarding their general experiences in Haiti. In addition, responders were asked to answer questions regarding specific elements of the support systems offered to them before, during, and after deployment. Finally, they were asked to reflect on the effectiveness of the existing systems of support, and identify any perceived gaps.

In accordance with Strauss and Corbin’s (1990) approach to grounded theory, the central phenomenon of this study was identified as the “need for supports for Canadian health and social service providers responding to the disaster in Haiti”.^4^^,^ 7 The following categories were used to structure the analysis and results of this grounded theory study:

- Contextual and Intervening Conditions: a description of the societal, organizational andpersonal contexts and conditions that were present during the response
- Causal Conditions: reasons driving for the need for supports;
- Strategies: systems or interventions implemented that influenced the responder’s ability to respond with resiliency to the context and conditions; and
- Consequences: the “outcomes from using the Strategies in relation to the Context and Conditions" (Creswell, p.65)^8^

**Figure:**
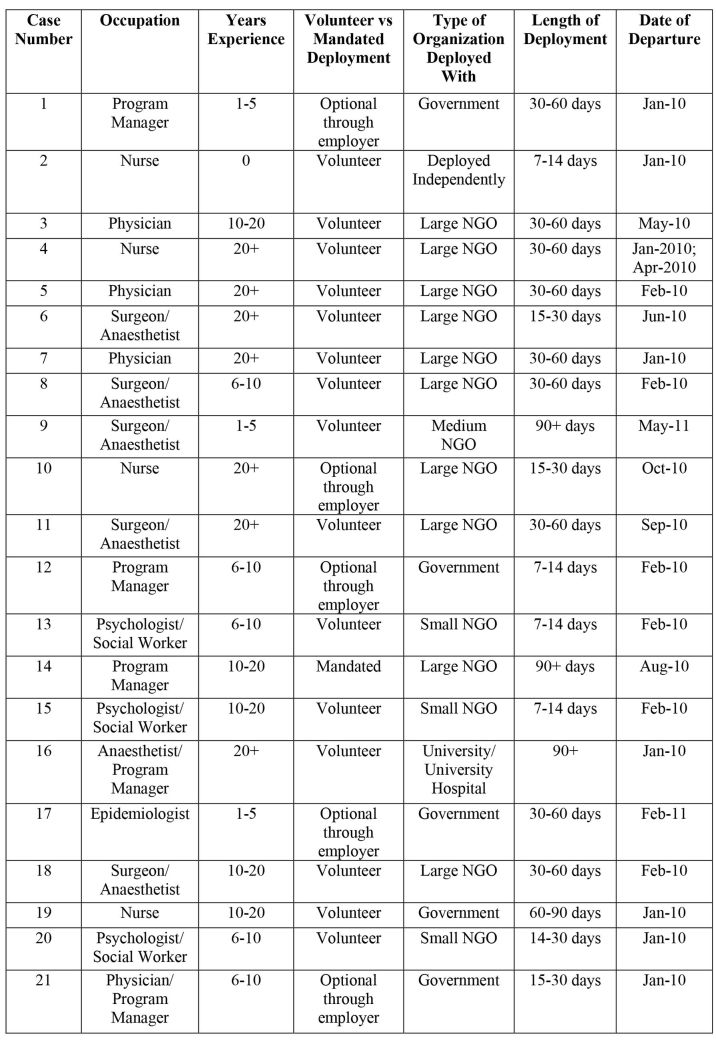
**Table 1. Participant Characteristics**

Questions pertaining to each of these categories were used to discover how available supports affected the experiences of front-line responders in Haiti. All of the interviews, researcher’s memos and reflections were transcribed, coded and analysed using NVivo9 qualitative software. The process included the development of higher level pattern codes to determine relationships between the identified categories. To remain consistent with structured grounded theory methodology 7 ^,^ 8, emergent themes were identified based on the categories surrounding the central phenomenon. To ensure saturation of the data was reached, the constant comparison method was used. 9 Analysis meetings involving all authors were regularly scheduled throughout the duration of the study, to ensure the processes of data collection and analysis remained objective and consistent. Triangulation using literature was used to re-evaluate the findings of this data, postanalysis. A previous study highlighting the participant findings has been previously published (Refer to Fahim et al., 2013 for additional thick descriptions of participant data).

## Results

In keeping with the style of Strauss and Corbin’s^7^ approach to grounded theory, the results for this study are presented in relation to the categories previously outlined. As shown in Figure 1: *Model of the Context, Strategies,* *Causes and Consequences of the Need for Supports for* *Canadian Health and Social Service Providers Responding* *to the Disaster in Haiti*, the contextual, causal and consequential conditions stem out of the central phenomenon of this study. As seen in the data, the Context of the response was a central feature of the aftermath of the earthquake.

**Model of the Context, Strategies, Causes and Consequences of the Need for Supports for Canadian Health and Social Service Providers Responding to the Disaster in Haiti:**
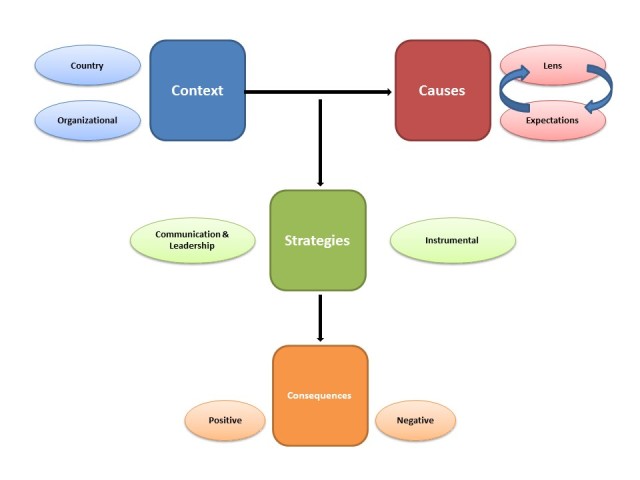

The first contextual factor, the country context, refers to the context within Haiti and the Haitian society at the time. Volunteers were faced with the challenge of performing emergency and medical procedures with little supplies, while coping with crumbling infrastructure, frequent tremors in the ground, and harsh conditions, such as extreme heat. Secondly, the context of the various relief organizations was explored to determine the organizational and coordinating factors that affected the response context. The participant interviews indicate that the organizational context not organized and was often described as a scene of ‘chaos’. As seen in Figure 1, the country and organizational context directly affected the ‘personal context’ of individuals responding to this disaster. The devastation in Haiti was vast and required a special type of volunteer who possessed the physical and emotional capacity to respond with resilience during such a disaster. An individual’s training, past experiences, attitudes and willingness to work in a team were all found to be crucial for their resiliency on-site. These elements are defined as the ‘causes’ of the central phenomenon, or the factors that influence and determine the types of supports required by the responders.

In reaction to the contexts, the Causes of the central phenomenon emerged and integrated with the need for supports along two main themes. The first cause for support related to unclear expectations, which led to feelings of uncertainty and confusion, fear, lack of communication and awareness, stress and guilt. These feelings stemmed from a variety of causes, including unclear roles on-site, disorganization within teams and between organizations, and insufficient safety protocols, training, and psychological supports. The ‘personal context’ of an individual, which we will later refer to as the ‘lens’, relates to participants’ perceptions that influence their experiences, attitudes and personal initiatives in preparation for deployment.

In response to the contexts and conditions, many organizations employed two main strategies to support their delegates. As seen in the model, the supports were determined based on both the context of the disaster as well as the causes. The first of these strategies, Instrumental Supports, includes safety and security supports, psychological and emotional supports, as well as physical supports. The second category of strategies focuses on Communication and Leadership. These supports include recruitment and screening techniques, creating positive team dynamics, appointing effective team leaders, ensuring clear and consistent communication between Canadian headquarters and the on-site base, and highlighting the need for accurate media coverage of the disaster situation, in order to avoid confusion and unclear expectations. There was no universal standard for supports given to response workers in Haiti. Therefore, responders were heavily reliant on their affiliated organizations to provide them with various types of support pre-departure, on-site and post-deployment. Results from the interviews indicate that the effectiveness of these supports varied greatly by organization.

Finally, the Consequences of the response refer to the overall experience and impact of the response, as perceived by the participants. The consequences can be positive or negative, and are determined as a result of an interaction between the context, an organization’s support strategy, and an individual’s expectations and personal lens. According to Strauss and Corbin^7^ , Consequences are used to determine the effects of the central phenomenon. In this study, the participant’s need for supports was mitigated by their own personal experiences, ultimately affecting their overall perception of the response. Through an exploration of the consequences, in relation to the other grounded theory categories, three main themes were derived and presented below for the purpose of this theoretical framework.

**(1) The “lens” can mediate the effectiveness of a support strategy**

The data indicate that a participant’s lens can positively or negatively mediate, either by adding to or taking away from, the effects of a support strategy on the desired outcome (i.e., an overall positive experience). The lens is shaped by background experiences, personal beliefs and attitudes, as well as individual initiatives and preparedness. Thus, achieving a positive experience requires the recruitment of delegates who possess a specific lens, or set of characteristics, making screening and recruitment a critical factor for all organizations. Many participants, particularly those stemming from smaller organizations, reported that there was no formal screening program required, before deploying as a volunteer. Participants agreed that organizations should screen for individuals who demonstrate ongoing interest in disaster relief, and who have taken personal steps, such as familiarizing themselves with a country’s language or completing emergency response training courses, in preparation for deployment. By doing so, organizations can effectively recruit during times of normalcy, thus bypassing the initial ‘disaster tourism’ or ‘response hype’ that accompanies large disasters. As stated by one participant,

“*The idea is that you recruit when there’s nothing traumatic going on, so that way you* *really have time to do training, answer questions, check out the background of* *volunteers...but when there’s been a big disaster, you’re always begging people that are* *like what we’d call maybe walk-up volunteers. And so you have to be prepared to try to* *do some kind of training or check out before they go.” (*Faith-based organization)

In addition to mediating supports, the lens can, in unique settings and situations, compensate for lack of supports or ineffective support strategies. This means that the lens can itself suffice, or can independently serve, as a support mechanism that is innate to the individual. While reactions such as these are ultimately attributed to an individual support mechanism, they can be affected, either positively or negatively, when in combination with support strategies. The data indicate that the perceived level of effectiveness of supports was highly dependent on an individual’s attitude, beliefs, personal background and experiences.

**(2) Expectations can override the effects of support strategies to affect the outcome**

The data indicate that expectations can override the effects of a support strategy and affect the overall outcome of the response experience. This point differs from item (1) above, in that expectations differ greatly from the lens. The lens is an innate attribute, unique to the individual, and rooted in personal experiences, attitudes and beliefs. Expectations, however, are not as fixated and are formed specific to a response or situation. According to our participants, expectations are greatly shaped through 1) Personal beliefs 2) Training and briefing supports and 3) Organizational and Media influence. Thus, expectations are derived both intrinsically (personal beliefs and stigmas) and extrinsically (through training, briefing, effective information dissemination, and messages relayed by relief organizations and media). Not surprisingly, the data shows that previous experience with disaster response plays a strong role in developing personal beliefs and expectations regarding future responses and availability of supports. Delegates who had previously deployed to Haiti, or had completed similar disaster relief missions, typically held a better understanding of their roles and responsibilities than novice volunteers. Thus, having previous experiences with disaster, shapes a piece of the lens. If organizations consistently employ support strategies that provide a delegate with positive experiences, then, over time, the delegate’s lens will be shaped positively, thus affecting their outlook regarding the disaster response. These effects can be reversed if support strategies are not provided and the delegates experience negative deployments, thus deterring them from participating in future responses.

Our data indicates that, in regards to extrinsic factors, training plays a large role in shaping expectations. In fact, 40% of our participants would have liked to receive better pre-departure or early on-site briefing to better prepare them for their deployment. Briefs regarding the political and economic climate of the country, roles and team dynamics, and medical concerns specific to the disaster response were requested by the participants. The participants also indicated that the media (which is not typically a factor considered in support strategies) plays a role in shaping participant experiences and expectations. Inaccurate media coverage causes delegates to enter a context under false expectations, which may impact their ability to respond once on-site. Furthermore, negative or pessimistic media may discourage delegates who just returned from a large-scale disaster, such as Haiti, and lead to feeling under-appreciated. Holding briefing sessions and following up with delegates post-disaster can mitigate some of these negative outcomes. The biggest difference between the lens and expectations in relation to the available supports is that expectations are formed on a short-term basis, and can be easily swayed with information through training, briefing and media, while the lens is more integrated and takes time to develop. Therefore, the lens is not easily changed or affected by information or training. However, the lens can be swayed based on an individual’s experiences, which can be shaped by the types of supports available during deployment. In essence, the supports will ultimately determine whether or not the individual continues to respond to disasters, and if they do, what their attitude towards the response will be. This point is especially pertinent to health and social service providers, who are typically volunteers, and not mandated by their professional organization to respond to disasters.

**(3) Supports (instrumental and communicative & leadership) should be used effectively to** **shape the lens and expectations and ensure an overall positive response**

According to the data, it is evident that overall experiences were greatly dependent on the types of supports that were provided by the responder’s affiliated organization. Those who deployed with these larger, more established organizations typically felt well-supported and understood they were part of a ‘bigger picture’, while those who deployed with smaller organizations felt the response to be disorganized. As stated by one of the participants,

“*Everybody [organizations] wants to show that they are there and they are the ones* *doing the work. They are well intentioned, there’s no question. But because it’s not* *coordinated, sometimes they can harm each other.”* (Bilateral non-state institution)

All of the aforementioned supports are key to ensuring overall positive outcomes. As seen in the data, those who reported overall positive experiences in Haiti typically had formal and communicative and leadership supports available to them (regardless of whether or not they chose to use them or found them effective). Those who were not provided with these supports either reported a negative experience or cited these supports as lacking. In addition to training, participants highlighted the importance of safety and psychological/emotional supports for an effective response. In a comparison of two quotes reporting the effectiveness of training supports, one can see how similar supports can be perceived differently, based on an individual’s lens and experience. As stated by the first participant,

*“I know volunteers...some of them were a little shocked at the level of security and* *wondered ‘why do we have so much security, is there something we need to be worried* *about here?’ And that got them more upset. And then some of them were really relieved* *to see all of the security.”* (Faith-based organization)

This statement is reflected in a quote by a second participant, who highlights seemingly comprehensive (transportation, local driver, radio, briefings and security protocols) supports, yet does not believe these to be adequate for ensuring feelings of safety and security.

*“That’s where I thought things were really lacking [in regards to safety supports]. It’s* *kind of crazy when you think about it, you’re just kind of given a truck and a driver and* *you go out and there’s a radio...and there’s a security officer that briefs you on security* *and you have orders to be indoors by nightfall. But other than that, you’re really kind of* *on your own there.”* (Canadian government)

These types of examples are found throughout our data. It seems that when protocols were explained and supports were organized (in this case, safety supports), volunteers typically felt safe. However, excessive protocols sometimes led to feelings of worry or confusion, as did a lack of briefing. Ultimately, however, the individual’s lens and experiences shaped the perceived effectiveness of supports.

## Discussion

According to Strauss and Corbin, grounded theory is developed through an identification of emergent themes within the Context, Causes, Strategies and Consequences. 7 In order to develop a theory, the interaction between the four categories was explored in relation to overall outcomes and experiences to determine the themes. Based on these themes, a theoretical model, outlining the theoretical relationships between factors that influence the experiences and outcomes in the disaster response, was constructed. Supports alone do not determine the overall outcome of participant experiences. Instead, they interact with causal factors (expectations and the lens) to play a crucial role in determining overall outcomes. By targeting the factors that affect the Causes, organizations can better ensure an overall positive experience for their delegates. This relationship is demonstrated below in Figure 2:* A model outlining the theoretical relationships between factors that influence the overall experiences and* *outcomes in the disaster response*.

**A model outlining the theoretical relationships between factors that influence the overall experiences and outcomes in the disaster response:**
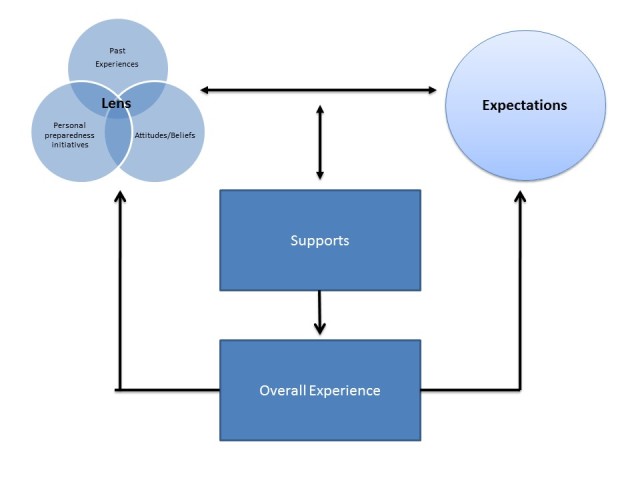

In order to understand the implications of this theory, it is important to consider the context in which this theory takes place. The context is especially important in Haiti, as it was challenging for both inexperienced delegates and organizations. According to a 2010 report created by the Pan American Health Organization (PAHO-WHO), there are four characteristics that created disadvantages for the response, which are as follows: “1) Haiti is a small country, among the poorest of the world, and had limited response capacity; 2) Haiti had weak institutions with little control over thousands of donor-supported NGOs; 3)Haiti had a lack of governance and a high level of corruption; and 4) Haiti had the absence of armed forces.” (de Ville de Goyet, Sarmiento, Grünewald of PAHO, p. 2.^10^ According to PAHO10, the earliest and most effective responders were individuals who were already in Haiti at the time of the disaster. The data indicate that participants who deployed with large, experienced NGOs reported being provided with the greatest number of, as well as the most effective, support systems. These delegates were often trained pre-departure, had their physical needs cared for by the organization, had clear safety and security guidelines, and comprehensive psychological and emotional supports in place.

In addition to ensuring that adequate supports can be provided to volunteers, organizations must place great emphasis on their screening programs for deployments. This can often times be challenging, particularly as recruiters experience a sense of urgency to deploy health and social service workers. Traditionally, organizations sought volunteers who demonstrated good organizational skills, a high emotional intelligence, and sense of compassion. 11 ^,^ 12 However, the literature suggests that many of the current approaches to recruitment and screening for international disasters may be ineffective. 13 ^,^ 14 ^,^ 15 Our study showed that recruiters found it difficult to assess whether volunteers would continue to demonstrate resiliency, under high-pressure environments in Haiti. As such, recruiting individuals with past experience with disasters, as well as individuals who had undertaken personal preparedness initiatives, such as learning the language of the country, became important, particularly in the initial phases of the disaster.

As seen in Figure 2, by selecting the ‘right’ individuals, organizations better their probability of recruiting individuals with a lens that fits with the supports that they are able to provide. In addition, organizations should provide their volunteers with pre-departure training, in order to ensure that their expectations are realistic. This training should define the context of the country, the extent of the disaster, the physical conditions in which the delegates will be working, as well as the specific roles, tasks and objectives of each volunteer. It should also provide volunteers with practical information and safety techniques regarding their roles on-site, and should make known to them any psychological and emotional supports that are available through the organization.

As such, it can be noted that the causal factors (the expectations and the lens) facilitate a feedback loop with the supports. As seen in Figure 2, when organizations effectively target the extrinsic factors that affect these causes, they are more likely to have their volunteers perceive their supports positively. Furthermore, as the efficiency and effectiveness of supports are improved, the lens and expectations of the participants will also change. It is an interaction between the expectations and lens, in addition to the available supports, that determine the overall experience of a delegate. Therefore, there is an interaction between extrinsic and intrinsic factors. Support strategies are extrinsic interventions aimed at assisting the delegates, while the lens and expectations are intrinsic support strategies, unique to each responder. Organizations must target both components to ensure an overall positive experience for their responders.

## Limitations

While a sample size of N=21 interviews is considered sufficient for the purpose of a grounded theory qualitative study, further research should be conducted to validate these findings within a larger population. Furthermore, it is important to remember that Haiti was a unique and extensive disaster, and data regarding the country context and conditions of a disaster should always be considered before developing support strategies. Regardless, all of the identified supports can be tailored to suit the particular context of the disaster-stricken country as well as the needs of responders.

## Competing Interests

The authors have declared that no competing interests exist.
